# Galectin-9 Mediates HIV Transcription by Inducing TCR-Dependent ERK Signaling

**DOI:** 10.3389/fimmu.2019.00267

**Published:** 2019-02-20

**Authors:** Florent Colomb, Leila B. Giron, Thomas A. Premeaux, Brooks I. Mitchell, Toshiro Niki, Emmanouil Papasavvas, Luis J. Montaner, Lishomwa C. Ndhlovu, Mohamed Abdel-Mohsen

**Affiliations:** ^1^Vaccine and Immunotherapy Center, The Wistar Institute, Philadelphia, PA, United States; ^2^Department of Tropical Medicine, Hawaii Center for AIDS, John A. Burns School of Medicine, University of Hawaii, Honolulu, HI, United States; ^3^GalPharma Co. Ltd., Takamatsu-shi, Takamatsu, Japan; ^4^Department of Immunology and Immunopathology, Kagawa University, Takamatsu, Japan

**Keywords:** HIV, lectins, galectin-9, T cell receptor, TCR signaling, rapamycin, HIV latency, HIV persistence

## Abstract

Endogenous plasma levels of the immunomodulatory carbohydrate-binding protein galectin-9 (Gal-9) are elevated during HIV infection and remain elevated after antiretroviral therapy (ART) suppression. We recently reported that Gal-9 regulates HIV transcription and potently reactivates latent HIV. However, the signaling mechanisms underlying Gal-9-mediated viral transcription remain unclear. Given that galectins are known to modulate T cell receptor (TCR)-signaling, we hypothesized that Gal-9 modulates HIV transcriptional activity, at least in part, through inducing TCR signaling pathways. Gal-9 induced T cell receptor ζ chain (CD3ζ) phosphorylation (11.2 to 32.1%; *P* = 0.008) in the J-Lat HIV latency model. Lck inhibition reduced Gal-9-mediated viral reactivation in the J-Lat HIV latency model (16.8–0.9%; *P* < 0.0001) and reduced both Gal-9-mediated CD4^+^ T cell activation (10.3 to 1.65% CD69 and CD25 co-expression; *P* = 0.0006), and IL-2/TNFα secretion (*P* < 0.004) in primary CD4^+^ T cells from HIV-infected individuals on suppressive ART. Using phospho-kinase antibody arrays, we found that Gal-9 increased the phosphorylation of the TCR-downstream signaling molecules ERK1/2 (26.7-fold) and CREB (6.6-fold). ERK and CREB inhibitors significantly reduced Gal-9-mediated viral reactivation (16.8 to 2.6 or 12.6%, respectively; *P* < 0.0007). Given that the immunosuppressive rapamycin uncouples HIV latency reversal from cytokine-associated toxicity, we also investigated whether rapamycin could uncouple Gal-9-mediated latency reactivation from its concurrent pro-inflammatory cytokine production. Rapamycin reduced Gal-9-mediated secretion of IL-2 (4.4-fold, *P* = 0.001) and TNF (4-fold, *P* = 0.02) without impacting viral reactivation (16.8% compared to 16.1%; *P* = 0.2). In conclusion, Gal-9 modulates HIV transcription by activating the TCR-downstream ERK and CREB signaling pathways in an Lck-dependent manner. Our findings could have implications for understanding the role of endogenous galectin interactions in modulating TCR signaling and maintaining chronic immune activation during ART-suppressed HIV infection. In addition, uncoupling Gal-9-mediated viral reactivation from undesirable pro-inflammatory effects, using rapamycin, may increase the potential utility of recombinant Gal-9 within the reversal of HIV latency eradication framework.

## Introduction

Antiretroviral therapy (ART) effectively suppresses HIV replication but does not achieve viral eradication due to the persistence of latently-infected, long-lived CD4^+^ T cells (1, 2). This persistent infection leads to continued immune activation, chronic inflammation, and ongoing damage to multiple organ systems (3, 4). Many studies indicate that HIV persistence is regulated, at least in part, by the immune system (5–8). Thus, understanding the host immune factors driving and maintaining HIV persistence is needed to develop new strategies to cure HIV and/or prevent the development of HIV-associated co-morbidities, which remain prevalent despite suppressive therapy.

One key regulator of immunological functions, and several cellular processes, is interactions between cell-surface glycans and glycan-binding proteins (lectins) (9–12). One class of lectins that play critical roles in T cell function are galectins, a family of β-galactoside-binding, soluble lectins (13–17). Among galectins, galectin-9 (Gal-9) has recently been recognized to play an essential role in regulating both adaptive and innate defense mechanisms and thus may be involved in HIV pathogenesis (18–20). Our prior work showed that endogenous secretion of Gal-9 is rapidly increased after HIV infection, and that elevated levels of Gal-9 do not return to normal after suppressive ART (20). More recently, we reported that the endogenous levels of Gal-9 are associated with HIV transcription *in vivo*, in plasma of HIV-infected, ART-suppressed individuals (8). We also demonstrated that treating CD4^+^ T cells with recombinant Gal-9 (rGal-9) induces HIV transcription and reverses HIV latency *in vitro* and *ex vivo* (8). However, the signaling pathways by which Gal-9 modulates HIV transcriptional activity remain unclear.

The goal of this work was to identify the signaling mechanisms underlying Gal-9-mediated HIV transcription. We hypothesized that Gal-9 modulates HIV transcriptional activity through T cell receptor (TCR) signaling transduction, based on results in non-HIV contexts showing that galectins, including Gal-9, modulate TCR-signaling (21–23), and that Gal-9 interacts with various cell-surface proteins known to induce TCR signaling, including CD44 (24) and 41-BB (25–27). We show that Gal-9 modulates HIV transcription through activating the TCR-downstream ERK and CREB signaling pathways in a lymphocyte-specific protein tyrosine kinase (Lck)-dependent manner. This signaling pathway also induces an undesirable, pro-inflammatory response, namely secretion of IL-2 and TNF-α, and activating CD4^+^ T cells. This pro-inflammatory response can be inhibited using the mTOR pathway inhibitor, rapamycin, without impacting Gal-9-mediated viral reactivation. Our results could have implications for understanding the role of endogenous galectin-9 in modulating TCR signaling *in vivo* and maintaining chronic immune activation during ART-suppressed HIV infection. In addition, uncoupling Gal-9-mediated viral reactivation from undesirable pro-inflammatory effects, using rapamycin, may increase the potential utility of rGal-9 within the reversal of HIV latency eradication framework.

## Materials and Methods

### Cell Lines

As a model of HIV latency, we used “J-Lat” cells, which harbor latent, transcriptionally competent HIV provirus that encodes green fluorescent protein (GFP) as an indicator of viral reactivation (28, 29). We have shown in our previous work (8) that this latency model mimics the impact of Gal-9 on HIV transcription *ex vivo* using CD4^+^ T cells from HIV-infected ART-suppressed individuals. Therefore, it can be used to investigate the signaling mechanisms underlying Gal-9-mediated viral transcription. J-Lat 5A8 clone was kindly provided by Dr. Warner Greene (The Gladstone Institute of Virology and Immunology). J-Lat 15.4 (catalog number 9850), 10.6 (catalog number 9849), and Jurkat E6-1 (catalog number 177) clones were provided by the NIH AIDS Reagent Program (Germantown, MD). J.CaM1.6 clone, a derivative mutant of Jurkat cells, which is deficient in Lck activity (30), was purchased from American Type Culture Collection (ATCC; Manassas, VA; catalog number CRL-2063). Cell lines were maintained in Roswell Park Memorial Institute (RPMI) medium with L-glutamine (Corning Cellgro, Tewksbury, MA, United States), supplemented with 10% Fetal Bovine Serum (Gibco, Thermo Fisher Scientific, Waltham, MA, United States), and 1% Penicillin/Streptomycin (Thermo Fisher Scientific, Waltham, MA, United States).

### Primary Cells

Cryopreserved peripheral blood mononuclear cells (PBMCs) were retrospectively collected from five HIV-infected individuals on suppressive ART, enrolled in the Philadelphia FIGHT cohort. Research protocols were approved by Wistar Institute and Philadelphia FIGHT Committees on Human Research. Written informed consent was obtained for all participants, and all data and specimens were coded to protect confidentiality. Subject characteristics are documented in Supplementary Table 1.

### Reagents

A stable form of recombinant galectin-9 was obtained through our collaborators at GalPharma Co., Ltd. (Kagawa, Japan). A natural form of recombinant galectin-9 was purchased from R&D Systems (Minneapolis, MN; catalog # 2045-GA-050). Lck inhibitor A770041 was purchased from Axon Med Chem (Reston, VA). ERK inhibitor LY3214996 was purchased from Selleck Chemicals (Houston, TX). CREB inhibitor 666-15 and InSolution Rapamycin were purchased from Millipore Sigma (Burlington, MA). SMARTpool Accell Lck siRNA was purchased from Dharmacon (Lafayette, CO; cat # E-003151-00-0005) and Silencer™ Select Negative Control No. 1 siRNA was purchased from Thermo Fisher (Waltham, MA; cat# 4390843). ImmunoCult Human CD3/CD28 T Cell Activator was purchased from STEMCELL (Vancouver, BC, Canada).

### Phosphorylated CD3ζ-Chain Quantification

J-Lat 5A8, Jurkat, or J.CaM1.6 cells were cultured at 1 × 10^6^ cells/ml and treated with stable form of rGal-9 (200 nM), natural form of rGal-9 (200 nM), or an equivalent volume of phosphate buffered saline (PBS), in the presence of Lck inhibitor (1 μM) or an equivalent volume of dimethyl sulfoxide (DMSO). After 15 min, cells were collected, washed twice in ice-cold PBS, fixed, and permeabilized using the BD Cytofix/Cytoperm kit according to the manufacturer's instructions. Fixed cells were stained with PE anti-CD247 (TCRζ, CD3ζ) antibody (Clone 6B10.2, Biolegend), washed, and analyzed for PE fluorescence using LSR II flow cytometer and FACSDiva software (Becton Dickinson, Mountain View, CA, United States). Data were analyzed using FlowJo (TreeStar Inc., Ashland, OR, United States).

### Human Phospho-Kinase Antibody Array

Proteome profiler human phospho-kinase arrays were purchased from R&D Systems, Inc. (Minneapolis, MN, United States). Ten million J-Lat 5A8 cells were treated for 30 min with 200 nM rGal-9 or an equivalent volume of PBS. Cells were washed twice in cold PBS and processed according to the manufacturer's instructions. Briefly, the cells were lysed for 30 min on a rotating shaker at 4°C. Lysates were centrifuged at 14,000 g for 5 min, and supernatants were incubated on the array membrane overnight on an orbital shaker at 4°C. Membranes were washed three times and incubated for 2 h at room temperature with anti-horseradish peroxidase antibody. After another three washes, membranes were incubated for 1 min with Chemi Reagent. Membrane images were captured using ImageQuant LAS 4010 (GE Healthcare Bio-Sciences, Pittsburg, PA, United States) and densitometry analysis was performed with Image Studio Lite Version 5.2 (LI-COR Biotechnology, Lincoln, NE, United States).

### Measurement of HIV Latency Reversal

J-Lat 5A8, 10.6, and 15.4 cells were pre-incubated for 1 h with 1 μM for Lck inhibitor, 1 μM for ERK1/2 inhibitor, 1 μM for CREB inhibitor, 5 μM for rapamycin, or an equivalent volume of PBS. Cells were then treated with rGal-9 (multiple concentrations), 25 μl of ImmunoCult human CD3/CD28 T Cell Activator, or an equivalent volume of PBS, for 24 h. Mean fluorescence intensity of HIV-encoded GFP expression was assessed using LSR II flow cytometer and FACSDiva software. Data were analyzed with FlowJo.

### Lck Silencing Using Small Interfering RNA (siRNA)

J-Lat 5A8 were resuspended in Nucleofector solution (Lonza) at 1 × 10^6^ cells/100 μL, in the presence of 1.5 μM of Silencer™ Select (non-target) negative control No. 1 siRNA (Thermo Fisher, Cat# 4390843) or SMARTpool Accell Lck siRNA (Dharmacon, Cat # E-003151-00-0005). Cells were nucleofected using Amaxa Nucleofector4D (Lonza), according to manufacturer protocol for Jurkat clone E6.1 nucleofection. Cells were then plated in 1 ml of RPMI supplemented with 10% FBS. After 48 h, cells were resuspended in fresh medium at 1 × 10^6^ cells/ml, plated in 96-well plates, and treated with rGal-9 (200 nM), or an equivalent volume of PBS, for 24 h. Mean fluorescence intensity of HIV-encoded GFP expression was assessed using LSR II flow cytometer and FACSDiva software. Data were analyzed with FlowJo.

### Isolation and Treatment of Primary CD4^+^ T Cells

CD4+ T cells were enriched from the cryopreserved PBMCs by negative selection using the EasySep Human CD4^+^ T Cell Enrichment Kit (Stemcell Technologies), according to the manufacturer's instructions. Primary CD4^+^ T cells were maintained in RPMI with L-glutamine supplemented with 20% FBS. Primary CD4^+^ T cells were pre-incubated with 1 μM for Lck inhibitor or 5 μM for rapamycin. Cells were then treated with 500 nM of rGal-9, ImmunoCult human CD3/CD28 T Cell Activator, or an equivalent volume of PBS, for 24 h. Cells were centrifuged for 5 min at 250 g, and cells and supernatants were collected separately.

### Measurement of CD4^+^ T Cells Viability and Activation

The surface expression of CD69 and CD25 markers of T-cell activation were measured using flow cytometry. Cells were stained with Zombie Aqua Fixable Viability Kit (Biolegend) and then stained with the following fluorescently-conjugated monoclonal antibodies: APC mouse anti-human CD3 (Clone UCHT1, Biolegend), V450 mouse anti-human CD4 (Clone RPA-T4, Biolegend), PE mouse anti-human CD69 (Clone FN50, Biolegend), PerCP-Cy5.5 mouse anti-human CD25 (Clone M-A251, Biolegend). Apoptosis was determined using Propidium iodide and Annexin V Pacific blue (Biolegend). anti-CD95 (1 ug/ml; clone E0S9.1; Biolegend) stimulation for 6 h was used as positive control for apoptosis. Cells were analyzed using LSR II flow cytometer and FACSDiva software. Data were analyzed with FlowJo software.

### Measurement of TNF-α and IL-2 Levels Using ELISA

Supernatants from J-Lat 5A8 and primary CD4^+^ T cells cultures treated or not with rGal-9, αCD3/αCD28, and inhibitors were collected. Levels of TNFα and IL-2 cytokines were quantified using human TNF and IL-2 DuoSet Elisa kits (R&D Systems, Inc., Minneapolis, MN) according to the manufacturer's instructions. Optical density was measured at 450 and 540 nm using Versa Max microplate reader. Data were analyzed using GraphPad Prism.

### Measurement of Cytokine Secretion Using Multiplex Luminex

Isolated CD4^+^ T cells from three HIV-infected ART-suppressed individuals were treated with 200 nM Gal-9, 500 nM Gal-9, or 0.5% DMSO as control for 4 h, 24 h, or 3 days. Culture supernatants were collected after 3 days and were processed according to recommended manufacture procedure with a Milliplex MAP Human High Sensitivity T Cell Panel (EMD Millipore, Billerica, Massachusetts) for GM-CSF, TNF-α, IL-13, IL-12 (p70), IL-10, IL-8, IL-7, IL-6, IL-5, IL-4, IL-2, IL-1b, and IFN-γ. Samples were acquired on a Luminex 200 (EMD Millipore). Samples were run in duplicate. The intra-assay CV% for analytes measured was <7%. The inter-assay CV% was <11%.

### Statistical Analysis

Two-tailed paired and unpaired *t*-tests were used for comparisons. All statistical analyses were conducted using GraphPad Prism release 7.0 (GraphPad Software, San Diego, CA, United States) and statistical significance was set at a *p*-value of 0.05.

## Results

### Gal-9 Induces Lck-Dependent T Cell Receptor ζ Chain (CD3ζ) Phosphorylation

**Figure 1:**
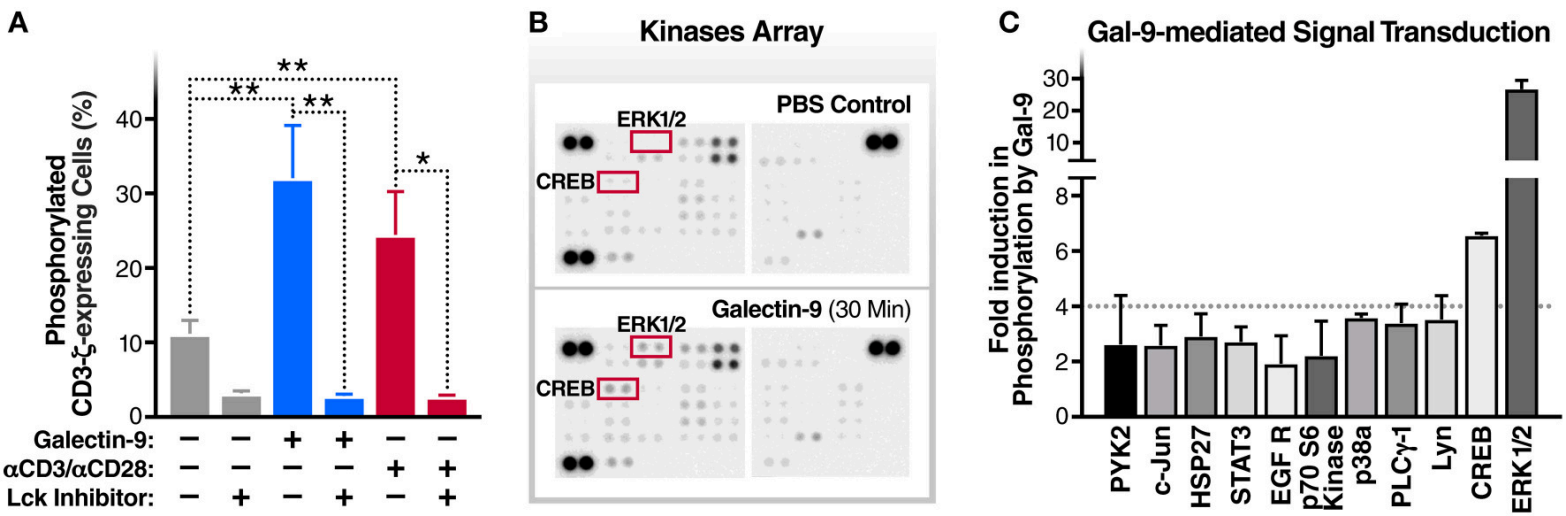
Gal-9 induces the phosphorylation of TCR complex CD3ζ chain, ERK, and CREB. **(A)** J-Lat 5A8 cells were treated with Gal-9 (200 nM, blue bars) or αCD3/αCD28 antibodies (red bars) for 15 min in the presence or absence of Lck inhibitor (1 μM) and stained with PE-conjugated anti-phospho-CD3ζ antibody. Cell staining/phosphorylation was quantified by flow cytometry. Mean ± SD is displayed, and statistical comparisons were performed using two-tailed unpaired *t*-tests. *p < 0.05, and **p < 0.01. **(B,C)** J-Lat 5A8 cells were treated with Gal-9 (200 nM) or an equivalent volume of PBS for 30 min and analyzed using Proteome Profiler Human Phospho-Kinase Array Kit. **(B)** Array images were captured using ImageQuant™ LAS 4,000 and **(C)** phosphorylation signal intensity was analyzed by pixel density quantification using Image Studio Lite.

The T cell receptor ζ chain (CD3ζ) is the principal signal transduction component of TCR signaling (31–33). Lck activity is required for the phosphorylation of signaling motifs in CD3-ζ and this phosphorylation is the initial step in the TCR signaling cascade (34, 35). To begin to test our hypothesis that Gal-9-mediates HIV transcriptional activity by activating TCR signaling, we examined the impact of rGal-9 on the phosphorylation of CD3ζ in the J-Lat 5A8 latency model. rGal-9 significantly induced the percentage of cells expressing phosphorylated CD3ζ [from 11.2 ± 1.8% to 32.1 ± 7.0% (mean ± SD); *P* = 0.0076] (Figure 1A). This induction was completely inhibited by preincubation with an Lck inhibitor (from 32.1 ± 7.0% to 2.8 ± 0.2%; *P* = 0.002) (Figure 1A). To confirm that Gal-9-mediated CD3ζ phosphorylation is Lck-dependent, we used the J.CaM1.6 clone (a derivative mutant of Jurkat cells, which is deficient in Lck activity). rGal-9 induced CD3ζ phosphorylation in wild type Jurkat cells [from 8.2 ± 2.2% to 44.4 ± 6.2% (mean ± SD); *P* = 0.0007], but had no effect on J.CaM1.6 (from 2.1 ± 0.6% to 1.6 ± 0.02%; *P* = 0.23) (Supplementary Figure 1). Confirming dose-dependent relationship, we found that rGal-9 is able to phosphorylate CD3ζ starting from 25 nM in the J-Lat 5A8 HIV latency model (Supplementary Figure 2). These data demonstrate that Gal-9 induces TCR-signaling in HIV latently-infected cells through Lck-mediated CD3ζ phosphorylation.

### Gal-9 Induces Phosphorylation of ERK and CREB Signaling Molecules

To identify components of the TCR signaling cascade that are induced by Gal-9 downstream of CD3ζ phosphorylation, we used Proteome Profiler Human Phospho-Kinase arrays to assess the phosphorylation levels of 43 kinases and related transcription factors. rGal-9 treatment of J-Lat 5A8 cells induced the phosphorylation of several downstream effectors of TCR signaling, including ERK1/2 (26.7-fold) and CREB (6.6-fold) (Figures 1B,C). These data demonstrate that Gal-9-mediated induction of CD3ζ phosphorylation results in activation of ERK1/2 and CREB signaling pathways.

### Gal-9 Modulates HIV Transcription Through Activating the TCR Downstream Signaling, ERK and CREB, in an Lck Dependent Manner

**Figure 2:**
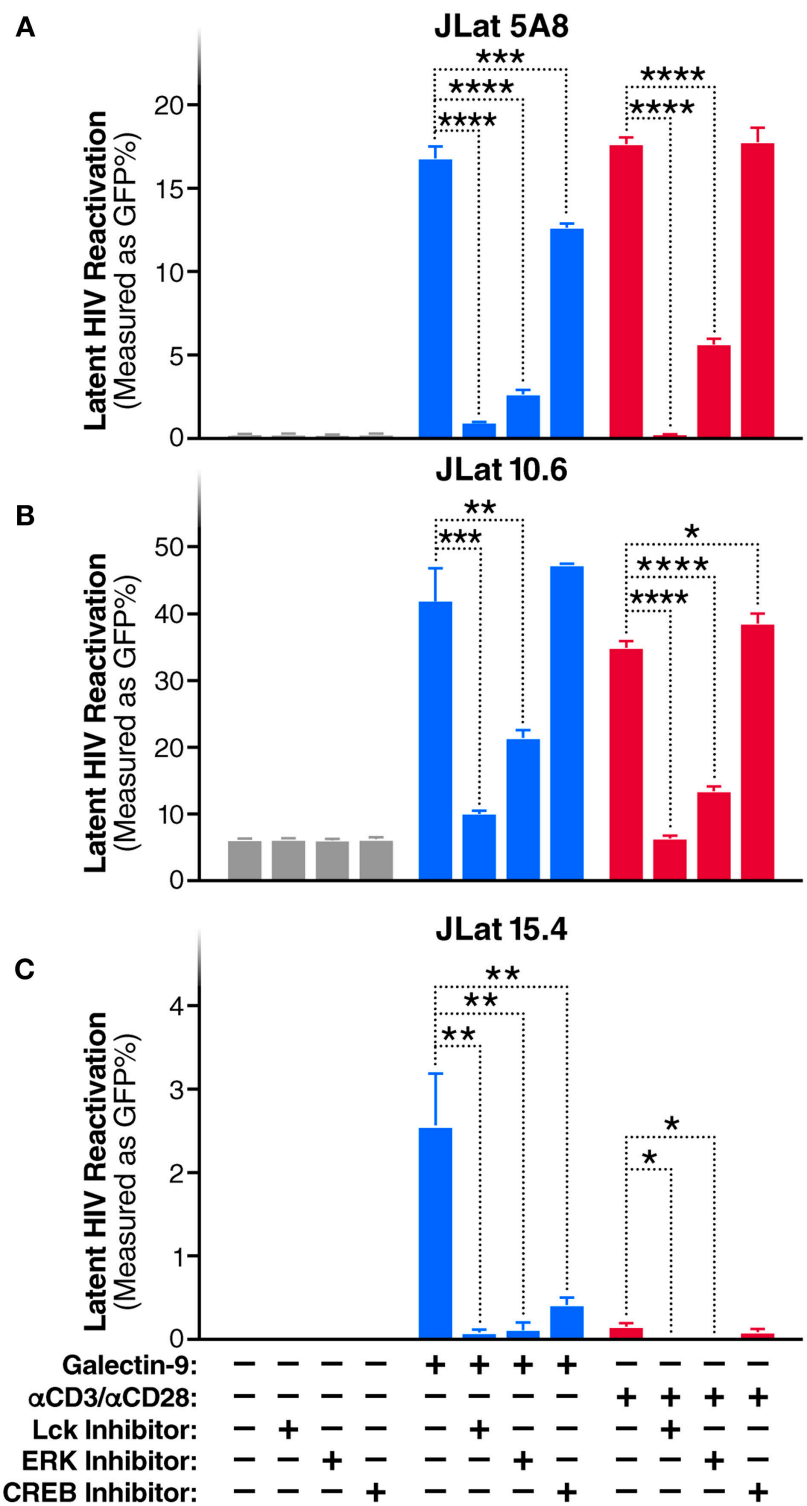
Gal-9 induces HIV transcription through TCR-dependent ERK/CREB signaling. Levels of latent HIV reactivation in three J-Lat clones 24 h after *in vitro* stimulation with Gal-9 (200 nM) in the presence or absence of 1 μM of Lck inhibitor, ERK inhibitor, or CREB inhibitor. HIV-encoded GFP expression was detected by flow cytometry. **(A)** J-Lat 5A8, **(B)** J-Lat 10.6, **(C) J**-Lat 15.4 cells. αCD3 /αCD28 antibodies (red bars) were used as a positive control. Mean ± SD is displayed, and statistical comparisons were performed using two-tailed unpaired *t*-tests. *p < 0.05; **p < 0.01, ***p < 0.001, and ****p < 0.0001.

To evaluate whether TCR/Lck-dependent ERK1/2-CREB phosphorylation modulate the reactivation of latent HIV by Gal-9, we assessed the impact of Lck, ERK, and CREB inhibitors on rGal-9-mediated HIV latency reactivation in three J-Lat HIV latency model clones, 5A8, 10.6, and 15.4. Lck inhibition strongly reduced rGal-9-mediated HIV latency reactivation in the J-Lat 5A8 clone [from 16.8 ± 0.7% to 0.9 ± 0.03% (mean ± SD); *P* < 0.0001], the J-Lat 10.6 clone (from 41.9 ± 4.9% to 9.9 ± 0.44%; *P* = 0.0004), and the J-Lat 15.4 clone (from 2.5 ± 0.64% to 0.1 ± 0.06%; *P* = 0.0027). ERK inhibition showed a similar strong reduction of rGal-9-mediated HIV latency reactivation in the J-Lat 5A8 clone [from 16.8 ± 0.7% to 2.6 ± 0.3% (mean ± SD); *P* < 0.0001], the J-Lat 10.6 clone (from 41.9 ± 4.9% to 21.23 ± 1.29%; *P* = 0.002), and the J-Lat 15.4 clone (from 2.5 ± 0.64% to 0.1 ± 0.1%; *P* = 0.0029). Last, CREB inhibition showed a modest, yet significant, reduction in rGal-9-mediated HIV latency reactivation in the J-Lat 5A8 clone [from 16.8 ± 0.7% to 12.6 ± 0.3% (mean ± SD); *P* = 0.0007], and the J-Lat 15.4 clone (from 2.5 ± 0.64% to 0.4 ± 0.1%; *P* = 0.0047) (Figures 2A–C). As expected, Lck/ERK/CREB inhibition had a similar impact on αCD3/αCD28-mediated HIV latency reactivation (Figures 2A–C). To confirm the role of Lck in Gal-9-mediated HIV latency reactivation, we used siRNA to silence Lck in the J-Lat 5A8 HIV latency model. Lck siRNA significantly reduced Gal-9-mediated HIV latency reactivation compared to non-target siRNA control [from 13.4 ± 0.3% to 6.8 ± 0.2% (mean ± SD); *P* < 0.0001] (Supplementary Figure 3). Confirming a dose-dependent relationship, we found that rGal-9 is able to reactivate a fraction of latent HIV at 10 nM in the J-Lat 5A8 HIV latency model (Supplementary Figure 4). Together, these data indicate that Gal-9 modulates HIV transcriptional activity through induction of TCR/Lck-mediated ERK1/2 signaling and to a lesser extent CREB signaling.

Throughout this study, we are using a stable form of galectin-9 (8, 22, 26, 36–39). To confirm that the natural form of Gal-9 functions in a similar way as the stable form in phosphorylating CD3ζ and reactivating HIV latency, we repeated some of the above experiments using a natural form of recombinant Gal-9 (R&D systems). The natural form of recombinant Gal-9 induced CD3ζ phosphorylation [from 8.2 ± 2.2% to 44.4 ± 6.2% (mean ± SD); *P* = 0.0007], and HIV latency reactivation [from 0.1 ± 00.6% to 20.2 ± 0.8% (mean ± SD); *P* < 0.0001] in the J-Lat 5A8 HIV latency model, and these effects were significantly inhibited by Lck inhibitor (Supplementary Figures 5A,B).

### Gal-9-Mediated CD4^+^ T Cell Activation Is Lck-Dependent

**Figure 3:**
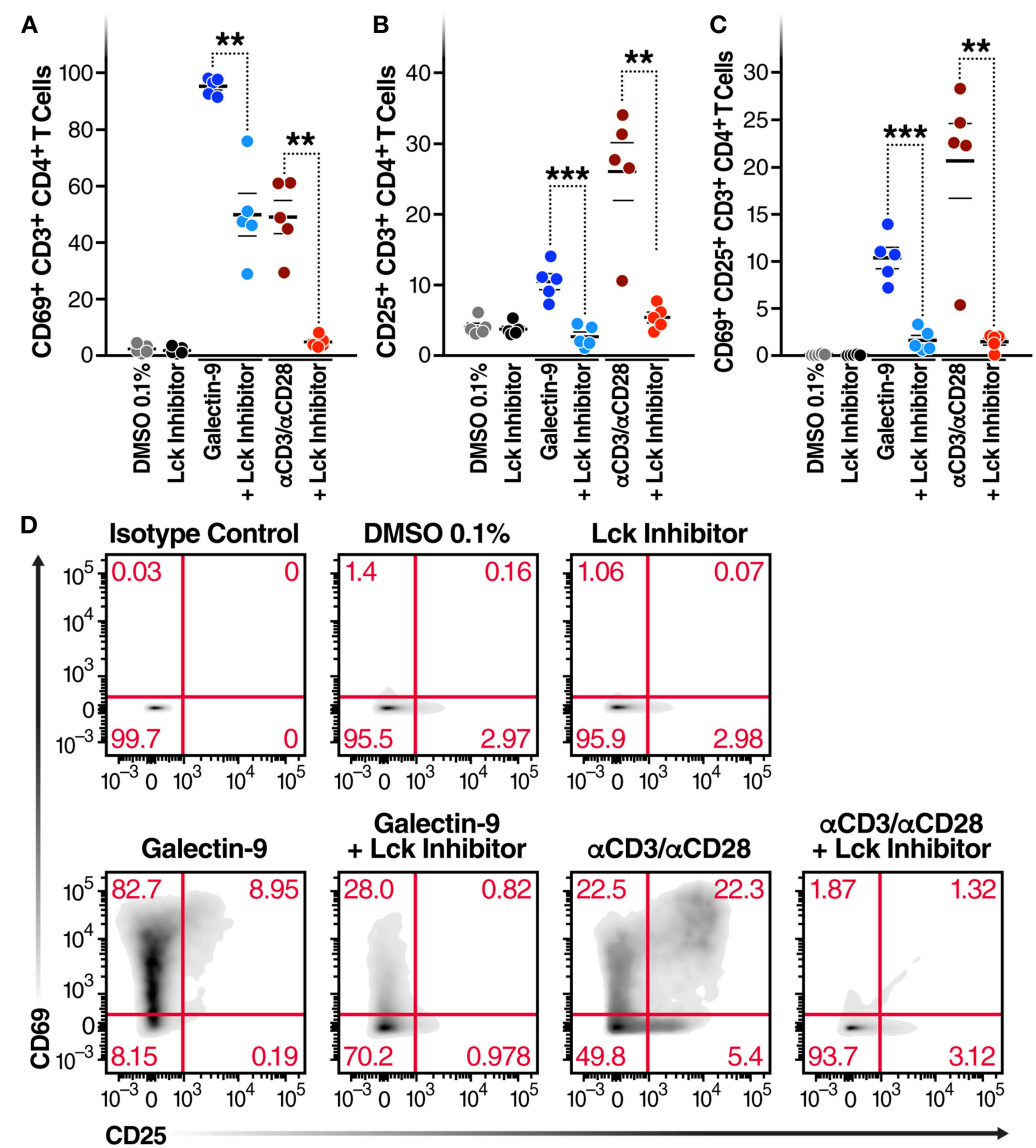
Gal-9-mediated CD4^+^ T cell activation is Lck-dependent. CD4^+^ T cells isolated from 5 HIV-infected ART-suppressed individuals were treated for 24 h with Gal-9 (500 nM) or the equivalent volume of PBS (Control) in the presence of 1 μM of Lck inhibitor or the equivalent volume of DMSO. Cells were analyzed by flow cytometry for expression of CD69 and CD25 activation markers. **(A)** % of CD4^+^ T cells expressing CD69, **(B)** % of CD4+ T cells expressing CD25, **(C)** % of CD4^+^ T cells co-expressing CD69 and CD25. **(D)** A representative flow cytometry plot of one individual. αCD3/αCD28 antibodies were used as a positive control. Mean ± SEM is displayed, and statistical comparisons were performed using two-tailed paired *t*-tests. **p < 0.01, and ***p < 0.001.

Next, to determine whether Gal-9-mediated T cell activation is dependent on the same mechanism as Gal-9-mediated viral activation, we examined the impact of Lck inhibition of Gal-9-mediated CD4^+^ T cell activation in primary cells from HIV-infected ART-suppressed individuals. Cell viability was determined using Zombie Aqua Fixable Viability and apoptosis was determined using Propidium iodide and Annexin V staining (Supplementary Figure 6). T cell activation was measured by co-expression of the T cell-surface activation markers CD69 and CD25. rGal-9 induced CD4^+^ T cell activation [from 0.14 ± 0.03% to 10.34 ± 1.1% (mean ± SEM)]. This effect was significantly reduced by Lck inhibition (from 10.34 ± 1.1% to 1.65 ± 0.5%; *P* = 0.0006) (Figure 3). Lck inhibition did not fully inhibit Gal-9-mediated T cell activation, suggesting that while TCR signaling plays a major role in Gal-9-mediated T cell activation, other signaling pathways may contribute to this effect.

### Gal-9 Induces IL2 and TNF-α Secretion by Inducing Lck-Dependent ERK and CREB Signaling

**Figure 4:**
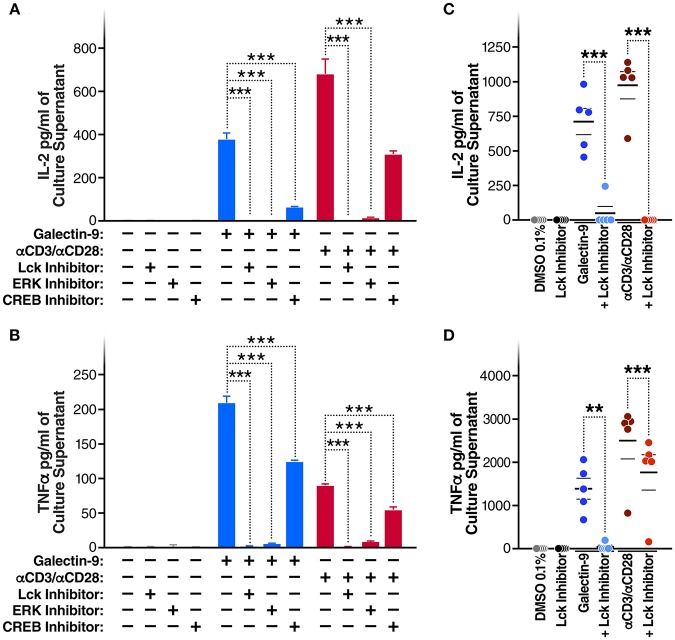
Gal-9 induces IL2 and TNF-α secretion by the same mechanism as viral reactivation. **(A)** IL2 and **(B)** TNF-α concentrations quantified by ELISA, in J-LAT 5A8 cell culture supernatants, after 24 h of Gal-9 treatment (200 mM) in presence or absence of 1 μM of Lck inhibitor, ERK inhibitor, or CREB inhibitor. Mean ± SD is displayed, and statistical comparisons were performed using two-tailed unpaired *t*-tests. ***p < 0.001. **(C)** IL2 and **(D)** TNF concentrations quantified by ELISA, in supernatants from CD4^+^ T cells from five HIV-infected, ART-suppressed individuals, after 24 h treatment with Gal-9 (500 nM) or the equivalent volume of PBS (Control) in the presence of 1 μM of Lck inhibitor or the equivalent volume of DMSO. αCD3/αCD28 antibodies were used as a positive control. Mean ± SEM is displayed, and statistical comparisons were performed using two-tailed paired *t*-tests. **p < 0.01, and ***p < 0.001.

Given that Gal-9 induces TCR signaling and activates T cells, we posited that the Gal-9-mediated reactivation of HIV latency would be accompanied by secretion of pro-inflammatory cytokines. Indeed, rGal-9 treatment of J-Lat 5A8 cells was associated with a significant induction of IL2 [from < 15.6 pg/ml to 375.6 ± 28.1 pg/ml (mean ± SD)] and TNFα (from <15.6 pg/ml to 208.8 ± 9.7 pg/ml). This effect was significantly reduced by inhibiting Lck, ERK, or CREB activity (*P* < 0.001; Figures 4A,B). Similar results were obtained using primary CD4^+^ T cells from HIV-infected, ART-suppressed individuals: rGal-9 significantly induced the production of IL2 (from <15.6 pg/ml to 700.2 ± 93.1 pg/ml (mean ± SEM)] and TNFα (from <15.6 pg/ml to 1372.68 ± 240.75 pg/ml); Lck inhibition significantly reduced IL2 (from 700.2 ± 93.1 pg/ml to 47.9 ± 47.9 pg/ml; *P* = 0.0006) and TNFα secretion (from 1372.68 ± 240.75 pg/ml to 37.8 ± 37.8 pg/ml; *P* = 0.0035) (Figures 4C,D). Further, we examined the impact of rGal-9 on the secretion of a panel of pro and anti-inflammatory cytokines. Supplementary Figure 7 shows that rGal-9 induces the secretion of several pro- and anti- inflammatory cytokines. Together the data in Figures 3, 4 demonstrate that Gal-9-mediated T cell activation and pro-inflammatory cytokine secretion are dependent on the same mechanism that induces HIV transcriptional activity, namely Lck-mediated induction of the TCR downstream ERK/CREB signaling. These undesirable, pro-inflammatory effects of Gal-9 limit its potential to be used as an HIV latency reversal agent. Thus, we asked whether it would be possible to separate the desirable (HIV latency reversal) from the undesirable (T cell activation and cytokine secretion) effects of Gal-9.

### Rapamycin Reduces Gal-9-Mediated Cytokine-Secretion Without Impacting the Ability of Gal-9 to Reactivate HIV

**Figure 5:**
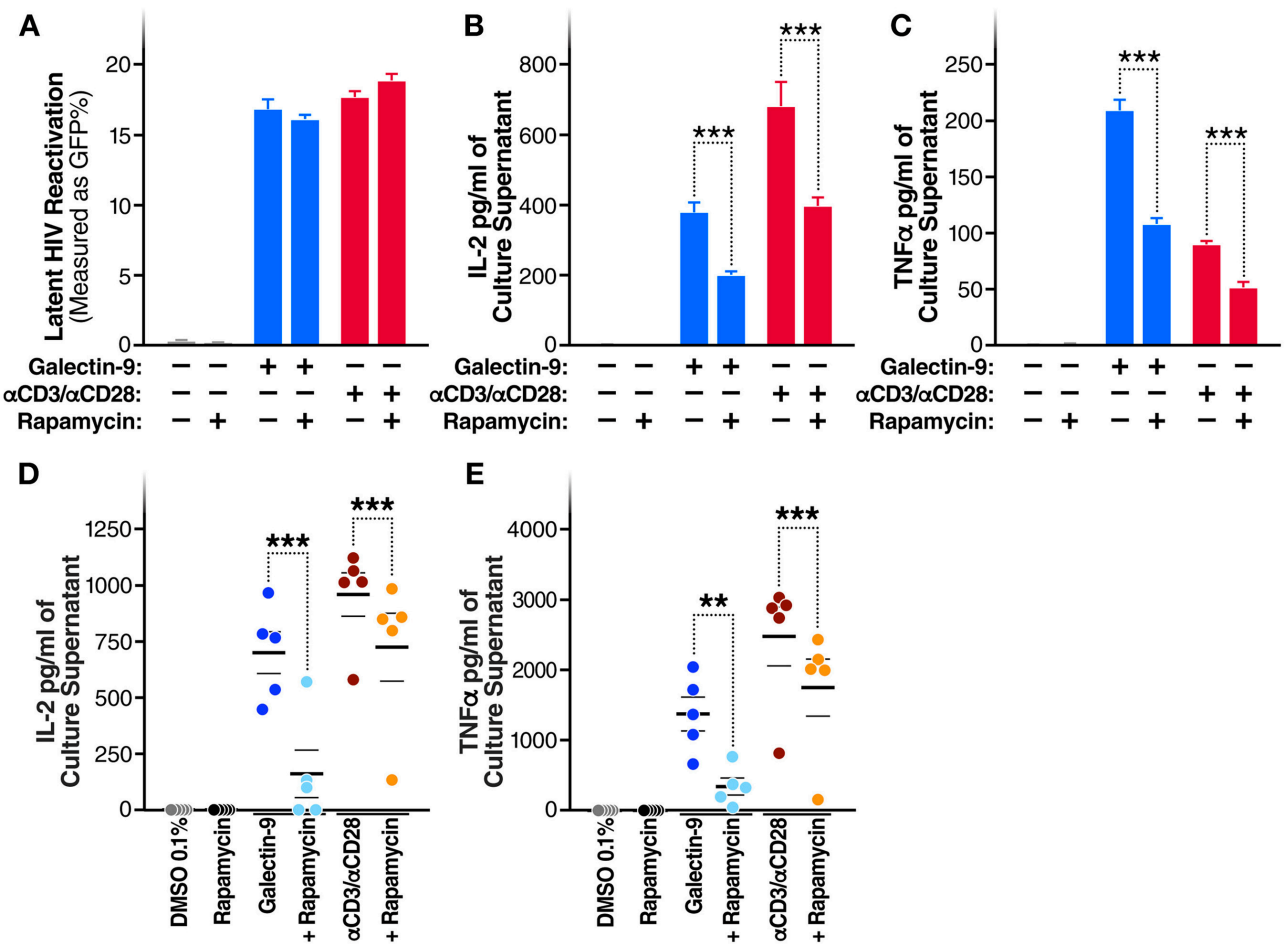
Rapamycin reduces Gal-9-mediated cytokine-secretion without impacting the ability of Gal-9 to reactivate HIV. **(A)** Viral reactivation in J-Lat 5A8 cells by Gal-9 (200 nM) in the presence or absence of 5 μM of rapamycin. Concentration of **(B)** IL2, and **(C)** TNF-α quantified by ELISA in cell culture supernatants. Mean ± SD is displayed, and statistical comparisons were performed using two-tailed unpaired *t*-tests. ***p < 0.001. Concentration of IL2 **(D)** and TNF-α **(E)** secretion quantified by ELISA in culture supernatants of CD4^+^ T cells isolated from five HIV+ ART-suppressed individuals after 24 h of Gal-9 stimulation (500 mM) in the presence of absence of 5 μM rapamycin. Mean ± SEM are displayed, and statistical comparisons were performed using two-tailed paired *t*-tests. **p < 0.01, and ***p < 0.001.

A recent study demonstrated that the immunosuppressive mTOR inhibitor rapamycin can uncouple HIV latency reversal from cytokine-associated toxicity (40). Furthermore, it has been shown that rapamycin can inhibit the proinflammatory effects of Gal-9 (41). We, therefore, investigated whether rapamycin could uncouple Gal-9-mediated latency reactivation from its concurrent effect on pro-inflammatory cytokine production. Co-treatment with rapamycin (5 μM) did not reduce the ability of Gal-9 to reactivate latent HIV (5 μM) [16.8 ± 0.7% compared to 16.1 ± 0.3% (mean ± SD); *P* = 0.2] (Figure 5A). However, rapamycin did significantly reduce Gal-9-mediated secretion of IL-2 [from 375.6 ± 28.13 pg/ml to 194.8 ± 12.43 pg/ml (mean ± SD); *P* < 0.001] and TNF (from 208.8 ± 9.7 pg/ml to 107.2 ± 5 pg/ml; *P* < 0.001) in the J-Lat 5A8 latency model (Figures 5B,C). Similarly, Rapamycin reduced Gal-9-mediated secretion of IL-2 [from 700.23 ± 91.1 pg/ml to 160.36 ± 105.9 pg/ml (mean ± SEM); *P* = 0.0006] and TNF (from 1372.68 ± 240.75 pg/ml to 340.13 ± 120.6 pg/ml; *P* = 0.0015) in primary CD4^+^ T cells from HIV-infected individuals on suppressive ART (Figures 5D,E). These data demonstrate that mTOR signaling inhibition may be used to uncouple Gal-9-mediated viral reactivation from undesirable pro-inflammatory effects.

## Discussion

Gal-9 promotes HIV transcription by a previously unidentified mechanism (8). Given that Gal-9 is known to cross-link several surface proteins, some of which are involved in TCR signaling and that Gal-9 can induce TCR signaling (22), we hypothesized that Gal-9 promotes HIV transcriptional activity through TCR signaling transduction. In this study, we demonstrate that Gal-9 modulates HIV transcription through activating the TCR-downstream ERK and CREB signaling pathways, in an Lck-dependent manner. We also show that this same signaling pathway that induces HIV transcription also induces an undesirable pro-inflammatory response, namely secretion of IL-2 and TNFα as a consequence of T cell activation. These undesirable, Gal-9-mediated pro-inflammatory responses can be inhibited using the mTOR pathway inhibitor, rapamycin, without blunting Gal-9-mediated viral reactivation.

Gal-9 has several effects on T cells during HIV infection. It renders CD4^+^ T cells less susceptible to HIV infection via induction of the host restriction factor cyclin-dependent kinase inhibitor 1 (p 21) (42). It can also increase HIV entry by inducing the CD4^+^ T cell-surface concentration of protein disulfide isomerase (PDI) (43). We have previously shown that the endogenous levels of Gal-9 are induced after HIV infection and that these levels do not return to normal levels after ART suppression (20). We also found a positive correlation between endogenous levels of Gal-9 and levels of HIV transcription in CD4^+^ T cells during ART suppression (8). The impact of this chronically elevated levels of Gal-9 on immune functions during ART-suppressed HIV infection is not clear. Our current study demonstrates that Gal-9 induces Lck-dependent ERK/CREB signaling in HIV-infected latently infected cells, which may explain the correlation between Gal-9 and HIV transcriptional activity *in vivo*. However, this induction of TCR signaling by Gal-9 raises an important question of whether elevated endogenous Gal-9 levels contribute to the state of chronic inflammation and chronic immune activation during suppressive ART. Elevated T cell activation persists during suppressive ART (44) and is associated with the development of HIV-associated co-morbidities and premature mortality (44–47); it may also contribute to HIV persistence (48–51). This chronic immune activation involves multifactorial mechanisms (52–59), and the persistent induction of TCR signaling by the elevated levels of endogenous Gal-9 may be playing an important, unrecognized, role in sustaining it. Interventions that target Gal-9 may prove useful in inhibiting chronic immune activation, which might ultimately reduce the development of HIV-associated co-morbidities and levels of HIV persistence during suppressive ART.

Gal-9 is a multifaceted lectin, with opposing roles in modulating innate and adaptive immune responses. Gal-9 was mainly described to exhibit immunosuppressive activities (24, 60–67). Gal-9 can also increase the function of regulatory T cells (T-regs) through interaction with CD44 (24) and may impair Natural Killer cells (NK) cytotoxicity and cytokine production through a Tim-3 independent mechanism (68). Along the same line, two recent studies demonstrated that Gal-9 suppresses B cell receptor signaling and B cell activation through interaction with CD45 and IgM-BCR complex (69, 70). However, other studies showed that Gal-9 exhibits immunopotentiating activity in the setting of immunosuppression (71) and induces TCR signaling (22), similar to our current study. Also, the endogenous levels of Gal-9 are induced during many inflammatory conditions (66, 72–74). Together, it is likely that Gal-9 effects are context-dependent and cell-type-dependent. The effect of Gal-9 on cell-mediated immunity in different subsets of T cells and other immune cells, during ART-suppressed HIV infection, warrants a broader investigation. Clarifying the signaling pathways induced or inhibited by Gal-9 in different cell-population, during HIV infection, may provide insights that may lead to the development of novel therapies to improve immune functionality, and reduce inflammation-associated co-morbidities, in the setting of viral suppression by ART.

The ability of Gal-9 to potently induce latent HIV transcription suggested that it could be considered within the “shock and kill” HIV eradication framework (8). However, the adverse Gal-9-mediated induction of pro-inflammatory cytokines, by the same pathway, limits the potential use of Gal-9 as a shock and kill agent. The recent study by Martin et al. (40) raised the possibility of using rapamycin, an immunosuppressive agent that does not affect TCR signaling, to prevent the adverse effects of T cell activation without impacting HIV transcription. Unlike several immunosuppressive agents that impact TCR signaling, rapamycin suppresses IL-2 downstream signaling (75, 76). Our data show that rapamycin is able to inhibit the pro-inflammatory impact of Gal-9 without affecting its ability to reactivate latent infection. That is in agreement with a previous study that used rapamycin to inhibit the proinflammatory effects of galectin-9 on dendritic cells and promote allograft tolerance in mice (41). Uncoupling Gal-9-mediated viral reactivation from undesirable pro-inflammatory effects, using rapamycin, may increase the potential utility of recombinant Gal-9 within the “reversal of HIV latency” eradication framework.

In summary, we identified TCR/Lck-dependent ERK1/2-CREB as the signaling pathways underlying Gal-9 modulation of HIV transcriptional activity. We also found that rapamycin can uncouple the Gal-9 impact on HIV transcription from the undesirable, pro-inflammatory secretions associated with inducing TCR signaling. Our data highlight the further investigations needed to comprehensively understand the immunologic consequences of Gal-9 *in vivo*, during ART-suppressed HIV infection. Our findings could have implications for understanding the role of endogenous galectin interactions in modulating TCR signaling and maintaining chronic immune activation, that persists during ART-suppressed HIV infection. Finally, uncoupling Gal-9-mediated viral reactivation from undesirable pro-inflammatory effects, using rapamycin, may increase the potential utility of recombinant Gal-9 within the reversal of HIV latency eradication framework.

## Author Contributions

FC, LG, TP, BM, EP, LM, LN, and MA-M designed and carried out experiments. EP and LM selected study subjects and provided samples. TN contributed reagents. FC, LG, TP, EP, TN, LM, LN, and MA-M analyzed and interpreted data. FC and MA-M wrote the manuscript, and all authors edited it.

### Conflict of Interest Statement

The authors declare that the research was conducted in the absence of any commercial or financial relationships that could be construed as a potential conflict of interest.

## Supplementary Material

Supplementary Figure 1 Gal-9-mediated CD3ζ phosphorylation is Lck-dependent. Jurkat E6-1 and J.CaM1.6 (a derivative mutant of Jurkat cells, which are deficient in Lck activity) cells were treated with Gal-9 (200 nM), or an equivalent volume of PBS, for 15 min and stained with PE-conjugated anti-phospho-CD3ζ antibody. Cell staining/phosphorylation was quantified by flow cytometry. Mean ± SD is displayed, and statistical comparisons were performed using two-tailed unpaired *t*-tests. ^***^*p* < 0.001.

Supplementary Figure 2 Low concentrations of Gal-9 induce CD3ζ phosphorylation. J-Lat 5A8 cells were treated with escalating doses of Gal-9 (0–200 nM) for 15 min and stained with PE-conjugated anti-phospho-CD3ζ antibody. Cell staining/phosphorylation was quantified by flow cytometry. Mean ± SD is displayed, and statistical comparisons were performed using two-tailed unpaired *t*-tests comparing each concentration to the 0 nM control. ^*^*p* < 0.05, and ^**^*p* < 0.01.

Supplementary Figure 3 Gal-9-mediated HIV latency reactivation is Lck-dependent. J-Lat 5A8 were transfected with Lck siRNA or non-target siRNA control using Amaxa Nucleofector4D. After 48 h, cells were treated with rGal-9 (200 nM), or an equivalent volume of PBS, for 24 h. HIV-encoded GFP expression was detected by flow cytometry. Mean ± SD is displayed, and statistical comparisons were performed using two-tailed unpaired *t*-tests. ^***^*p* < 0.001, and ^****^*p* < 0.0001.

Supplementary Figure 4 Low concentrations of Gal-9 reactivate latent HIV in the J-Lat HIV latency model. J-Lat 5A8 cells were treated with escalating doses of Gal-9 (0–200 nM) for 24 h. HIV-encoded GFP expression was detected by flow cytometry. Mean ± SD is displayed, and statistical comparisons were performed using two-tailed unpaired *t*-tests comparing each concentration to the 0 nM control. ^*^*p* < 0.05, ^***^*p* < 0.001, and ^****^*p* < 0.0001.

Supplementary Figure 5 The natural form of Gal-9 phosphorylates CD3ζ and reactivates latent HIV in Lck dependent. **(A)** J-Lat 5A8 cells were treated with the natural form of Gal-9 (200 nM) or an equivalent volume of PBS for 15 min and stained with PE-conjugated anti-phospho-CD3ζ antibody. Cell staining/phosphorylation was quantified by flow cytometry. **(B)** J-Lat 5A8 cells were treated with the natural form of Gal-9 (200 nM) or an equavelent volume of PBS for 24 h. HIV-encoded GFP expression was detected by flow cytometry. Mean ± SD is displayed, and statistical comparisons were performed using two-tailed unpaired *t*-tests. ^****^*p* < 0.0001.

Supplementary Figure 6 Impact of Gal-9 on CD4+ T cell viability and apoptosis. **(A)** CD4^+^ T cells isolated from 5 HIV-infected ART-suppressed individuals were treated for 24 h with Gal-9 (500 nM) or DMSO Control in the presence of 1 μM of Lck inhibitor or the equivalent volume of DMSO. Cell viability was determined using Zombie Aqua Fixable Viability staining. **(B)** A representative flow cytometry plot from one individual. **(C)** CD4^+^ T cells isolated from one HIV-infected ART-suppressed individual were treated for 24 h with Gal-9 (500 nM) or DMSO Control. Apoptosis was determined using Propidium iodide and Annexin V Pacific blue (Biolegend). anti-CD95 (1 ug/ml) stimulation for 6 h was used as positive control. Experiment was performed in duplicates. Mean ± SD is displayed **(D)** A representative flow cytometry plot of one replicate.

Supplementary Figure 7 Gal-9 induces the secretion of several pro- and anti-inflammatory cytokines. CD4^+^ T cells isolated from 3 HIV-infected ART-suppressed individuals were treated for 24 h with Gal-9 (200 nM), rGal-9 (500 nM), or DMSO Control for 4 h, 24 h, or 3 days. Culture supernatants were collected on day 3 and levels the 13 indicated pro- and anti-inflammatory cytokines were determined using Luminex assay.

Supplementary Table 1 Subject characteristics.
