# Supplementary figures and images for: Galectin-9 Mediates HIV Transcription by Inducing TCR-Dependent ERK Signaling
Source: Front Immunol. 2019 Feb 20;10:267. doi: 10.3389/fimmu.2019.00267 (PMC6391929; doi:10.3389/fimmu.2019.00267)

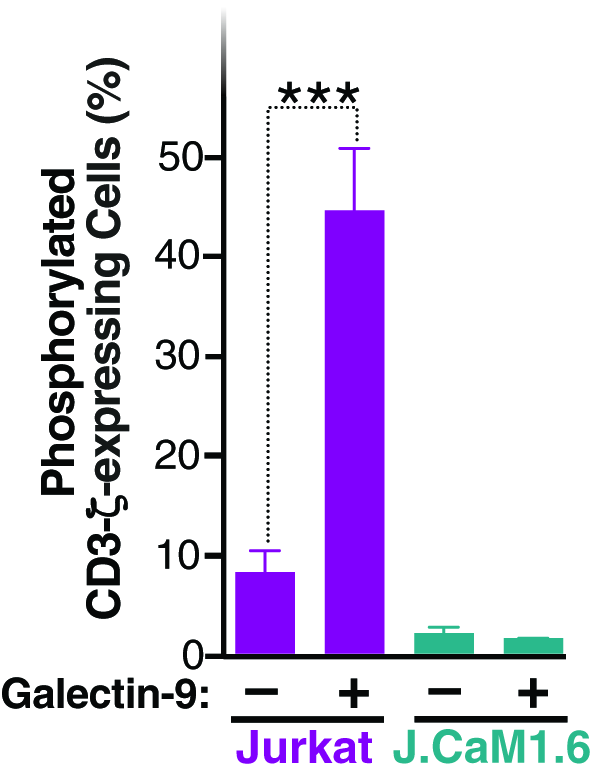

Supplement: Supplementary Figure 1 — Gal-9-mediated CD3ζ phosphorylation is Lck-dependent. Jurkat E6-1 and J.CaM1.6 (a derivative mutant of Jurkat cells, which are deficient in Lck activity) cells were treated with Gal-9 (200 nM), or an equivalent volume of PBS, for 15 min and stained with PE-conjugated anti-phospho-CD3ζ antibody. Cell staining/phosphorylation was quantified by flow cytometry. Mean ± SD is displayed, and statistical comparisons were performed using two-tailed unpaired t-tests. ***p < 0.001. [file Image_1.TIF]

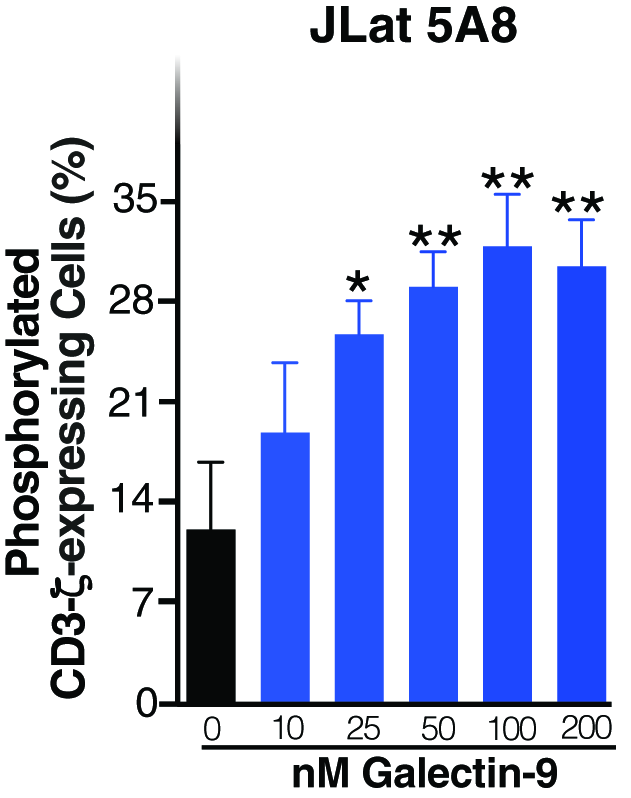

Supplement: Supplementary Figure 2 — Low concentrations of Gal-9 induce CD3ζ phosphorylation. J-Lat 5A8 cells were treated with escalating doses of Gal-9 (0–200 nM) for 15 min and stained with PE-conjugated anti-phospho-CD3ζ antibody. Cell staining/phosphorylation was quantified by flow cytometry. Mean ± SD is displayed, and statistical comparisons were performed using two-tailed unpaired t-tests comparing each concentration to the 0 nM control. *p < 0.05, and **p < 0.01. [file Image_2.TIF]

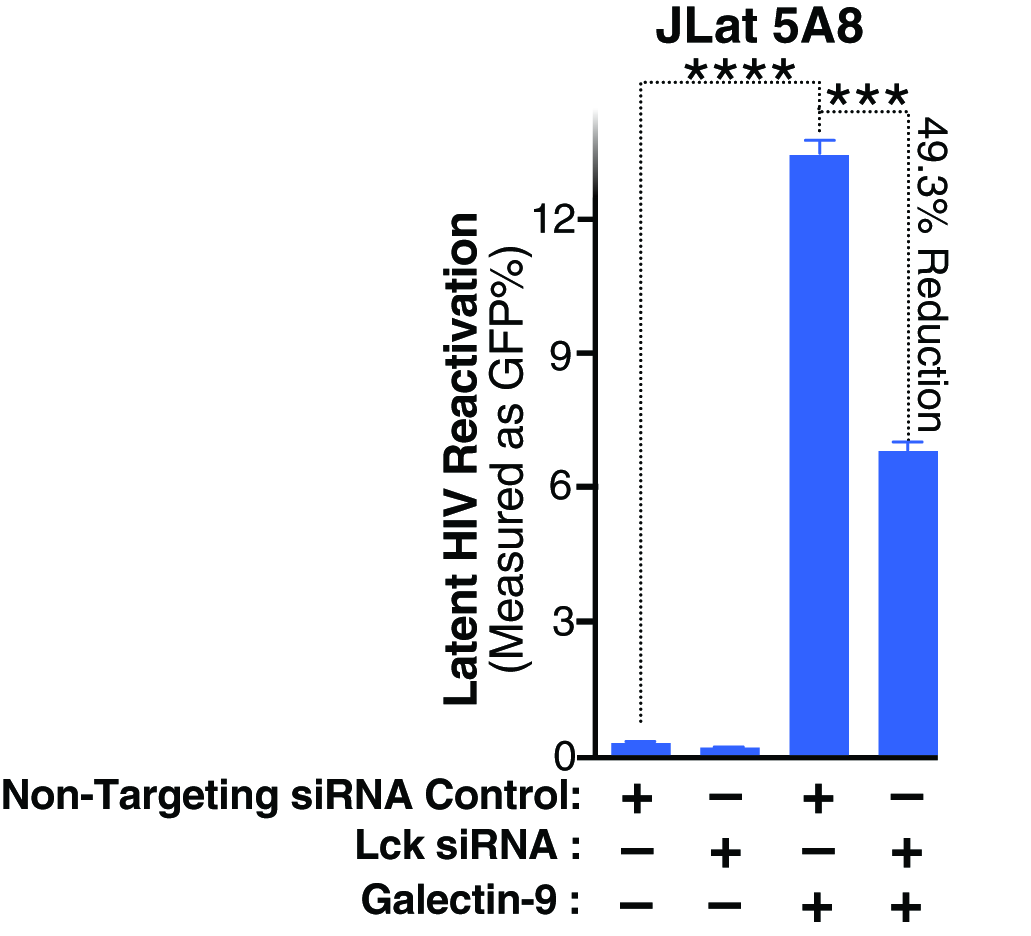

Supplement: Supplementary Figure 3 — Gal-9-mediated HIV latency reactivation is Lck-dependent. J-Lat 5A8 were transfected with Lck siRNA or non-target siRNA control using Amaxa Nucleofector4D. After 48 h, cells were treated with rGal-9 (200 nM), or an equivalent volume of PBS, for 24 h. HIV-encoded GFP expression was detected by flow cytometry. Mean ± SD is displayed, and statistical comparisons were performed using two-tailed unpaired t-tests. ***p < 0.001, and ****p < 0.0001. [file Image_3.TIF]

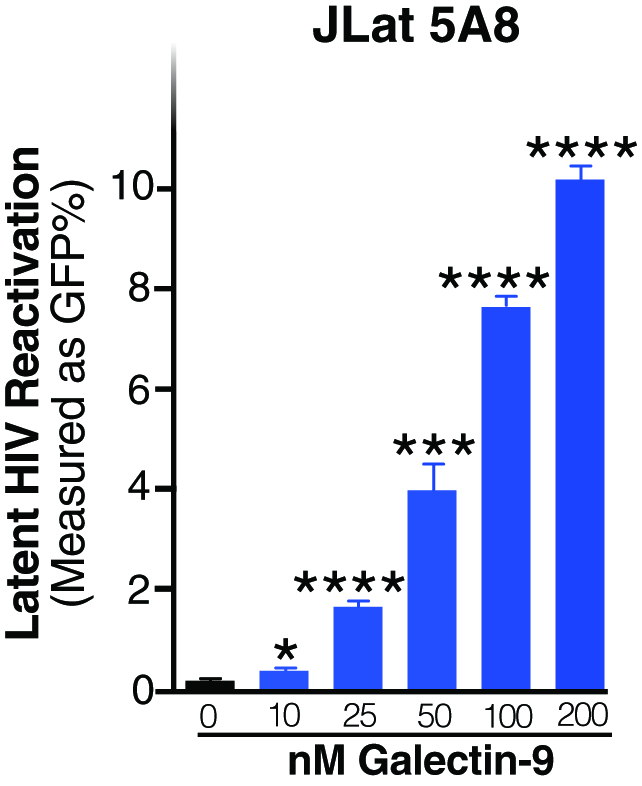

Supplement: Supplementary Figure 4 — Low concentrations of Gal-9 reactivate latent HIV in the J-Lat HIV latency model. J-Lat 5A8 cells were treated with escalating doses of Gal-9 (0–200 nM) for 24 h. HIV-encoded GFP expression was detected by flow cytometry. Mean ± SD is displayed, and statistical comparisons were performed using two-tailed unpaired t-tests comparing each concentration to the 0 nM control. *p < 0.05, ***p < 0.001, and ****p < 0.0001. [file Image_4.TIF]

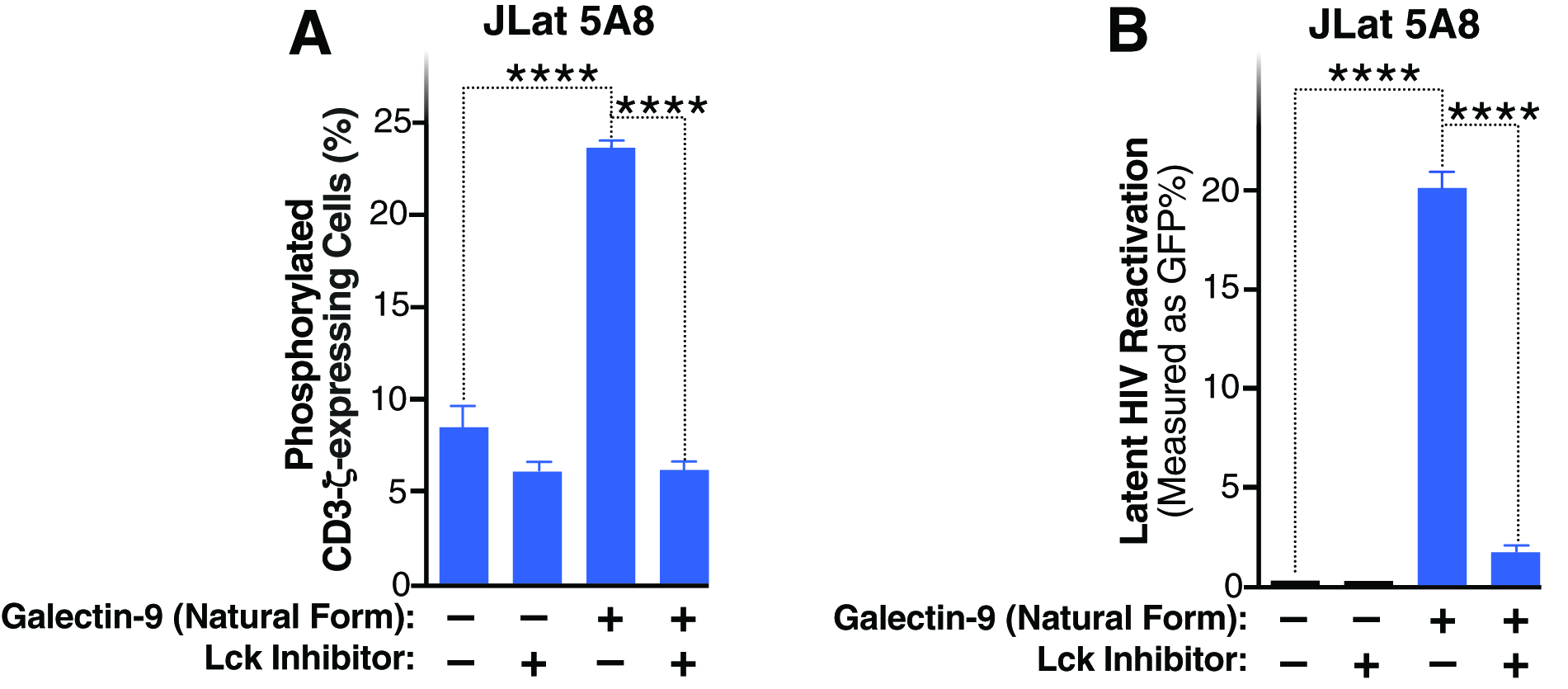

Supplement: Supplementary Figure 5 — The natural form of Gal-9 phosphorylates CD3ζ and reactivates latent HIV in Lck dependent. (A) J-Lat 5A8 cells were treated with the natural form of Gal-9 (200 nM) or an equivalent volume of PBS for 15 min and stained with PE-conjugated anti-phospho-CD3ζ antibody. Cell staining/phosphorylation was quantified by flow cytometry. (B) J-Lat 5A8 cells were treated with the natural form of Gal-9 (200 nM) or an equavelent volume of PBS for 24 h. HIV-encoded GFP expression was detected by flow cytometry. Mean ± SD is displayed, and statistical comparisons were performed using two-tailed unpaired t-tests. ****p < 0.0001. [file Image_5.TIF]

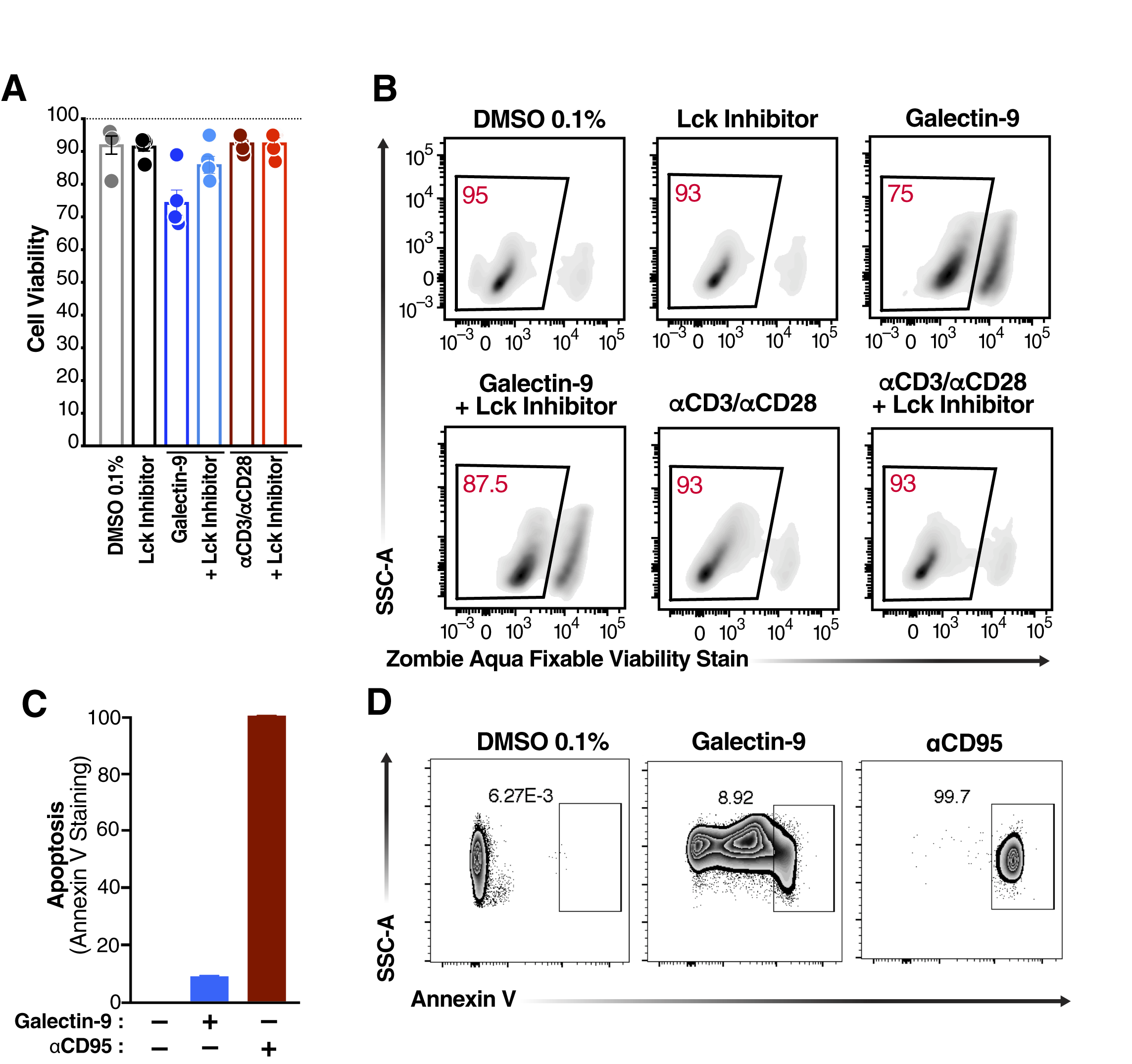

Supplement: Supplementary Figure 6 — Impact of Gal-9 on CD4+ T cell viability and apoptosis. (A) CD4+ T cells isolated from 5 HIV-infected ART-suppressed individuals were treated for 24 h with Gal-9 (500 nM) or DMSO Control in the presence of 1 μM of Lck inhibitor or the equivalent volume of DMSO. Cell viability was determined using Zombie Aqua Fixable Viability staining. (B) A representative flow cytometry plot from one individual. (C) CD4+ T cells isolated from one HIV-infected ART-suppressed individual were treated for 24 h with Gal-9 (500 nM) or DMSO Control. Apoptosis was determined using Propidium iodide and Annexin V Pacific blue (Biolegend). anti-CD95 (1 ug/ml) stimulation for 6 h was used as positive control. Experiment was performed in duplicates. Mean ± SD is displayed (D) A representative flow cytometry plot of one replicate. [file Image_6.TIF]

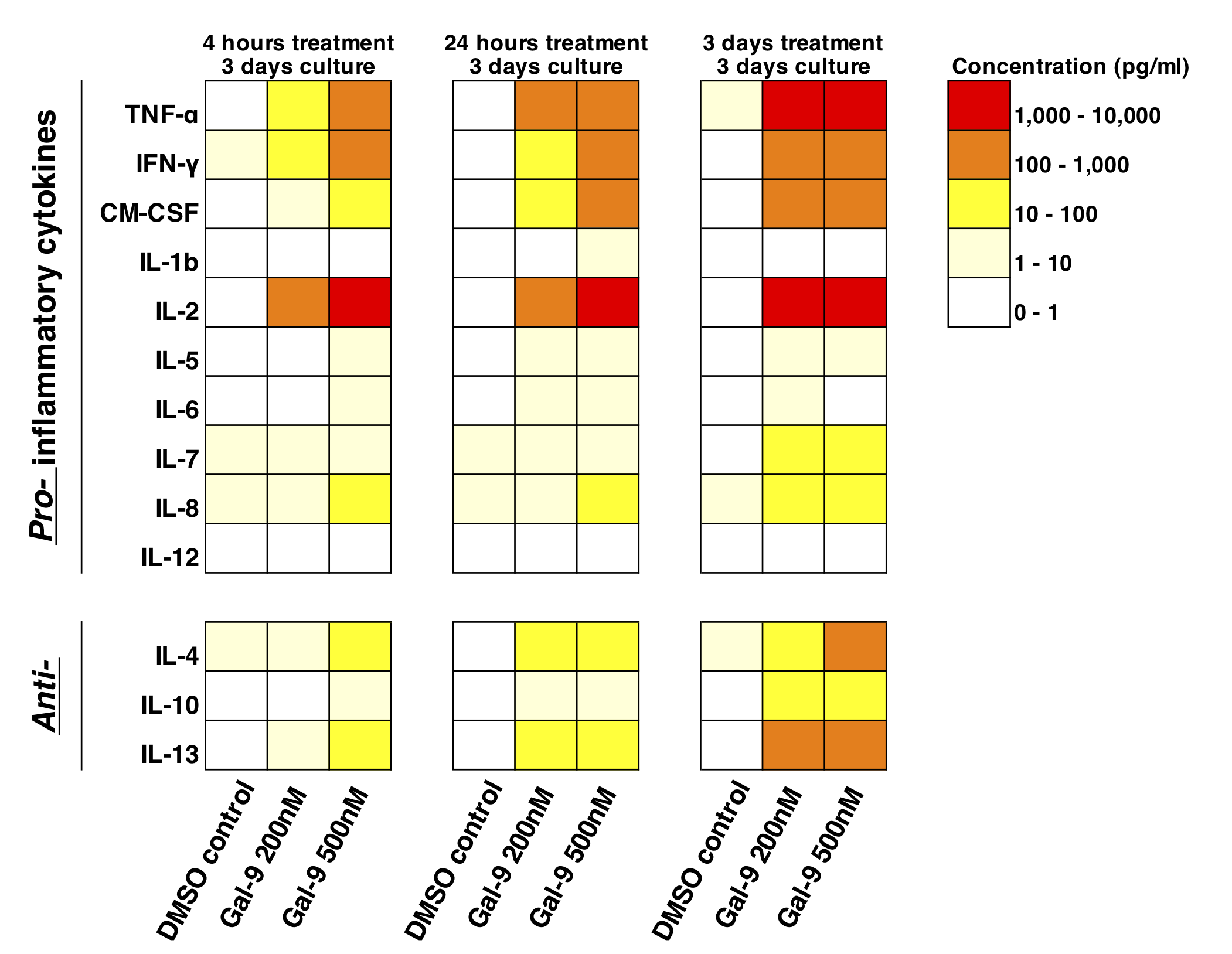

Supplement: Supplementary Figure 7 — Gal-9 induces the secretion of several pro- and anti-inflammatory cytokines. CD4+ T cells isolated from 3 HIV-infected ART-suppressed individuals were treated for 24 h with Gal-9 (200 nM), rGal-9 (500 nM), or DMSO Control for 4 h, 24 h, or 3 days. Culture supernatants were collected on day 3 and levels the 13 indicated pro- and anti-inflammatory cytokines were determined using Luminex assay. [file Image_7.TIFF]
