# Supplementary material for: Galectin-9 Mediates HIV Transcription by Inducing TCR-Dependent ERK Signaling
Source: Front Immunol. 2019 Feb 20;10:267. doi: 10.3389/fimmu.2019.00267 (PMC6391929; doi:10.3389/fimmu.2019.00267)
Supplement: Supplementary Table 1 — Subject characteristics. [file Table_1.docx]

Supplementary Material

**Galectin-9 Mediates HIV Transcription by Inducing TCR-dependent ERK Signaling**

Florent Colomb, Leila B. Giron, Thomas Premeaux, Brooks Mitchell, Toshiro Niki, Emmanouil Papasavvas, Luis J Montaner, Lishomwa Ndhlovu, Mohamed Abdel-Mohsen^*^

*** Correspondence:** Mohamed Abdel-Mohsen, Ph.D. Assistant Professor, The Wistar Institute, Philadelphia, PA, USA, 19104, E-mail: [mmohsen@wistar.org](mailto:mmohsen@wistar.org)

| **Supplementary Table 1.** Subject characteristics | | | | | | | | | | | |
| --- | --- | --- | --- | --- | --- | --- | --- | --- | --- | --- | --- |
| **Subject ID** | **YOB** | **Baseline age (years)** | **Sex** | **Ethnicity** | **Race** | **Drug regimen** | **Drug regimen class** | **Days on drug regimen** | **Baseline CD4 (cells/mm^3^)** | **Baseline CD4 (%)** | **Baseline plasma HIV-1 RNA (copies/ml)** |
| BEAT-018 | 1962 | 53 | M | Not Hispanic or Latino | Black | emtricitabine/tenofovir disoproxil fumarate/rilpivirine | NRTI/NRTI/NNRTI | 474 | 634 | 49 | <20 |
| BEAT-028 | 1981 | 35 | M | Hispanic or Latino | Other | emtricitabine/tenofovir disoproxil fumarate/rilpivirine | NRTI/NRTI/NNRTI | 611 | 810 | 48 | <20 |
| BEAT-039 | 1971 | 45 | M | Not Hispanic or Latino | Black | emtricitabine/tenofovir/atazanavir/ritonavir | NRTI/NRTI/PI/PI | 1932 | 771 | 37 | <20 |
| BEAT-047 | 1992 | 25 | M | Not Hispanic or Latino | Black | emitricitabine/elvitegravir/cobicistat | NRTI/INSTI/Pharmacokinetic enhancer (CYP3A inhibitor) | 80 | 547 | 31 | <20 |
| BEAT-048 | 1977 | 40 | M | Not Hispanic or Latino | Black | emtricitabine/tenofovir disoproxil fumarate/elvitegravir/cobicistat | NRTI/NRTI/INSTI/Pharmacokinetic enhancer (CYP3A inhibitor) | 614 | 550 | 26 | <20 |

YOB: Year of Birth; M; Male; NRTI: nucleoside reverse transcriptase inhibitor; NNRTI: non-nucleoside reverse transcriptase inhibitor; PI: protease inhibitor; INSTI: integrase strand transfer inhibitor; CYP3A: cytochrome P4503A
